# Live Cell Imaging During Germination Reveals Dynamic Tubular Structures Derived from Protein Storage Vacuoles of Barley Aleurone Cells

**DOI:** 10.3390/plants3030442

**Published:** 2014-09-05

**Authors:** Verena Ibl, Eva Stoger

**Affiliations:** Department for Applied Genetics and Cell Biology, Molecular Plant Physiology and Crop Biotechnology, University of Natural Resources and Life Sciences, Muthgasse 18, Vienna 1190, Austria; E-Mail: verena.ibl@boku.ac.at

**Keywords:** barley, aleurone, endosperm, PSV, germination

## Abstract

The germination of cereal seeds is a rapid developmental process in which the endomembrane system undergoes a series of dynamic morphological changes to mobilize storage compounds. The changing ultrastructure of protein storage vacuoles (PSVs) in the cells of the aleurone layer has been investigated in the past, but generally this involved inferences drawn from static pictures representing different developmental stages. We used live cell imaging in transgenic barley plants expressing a TIP3-GFP fusion protein as a fluorescent PSV marker to follow in real time the spatially and temporally regulated remodeling and reshaping of PSVs during germination. During late-stage germination, we observed thin, tubular structures extending from PSVs in an actin-dependent manner. No extensions were detected following the disruption of actin microfilaments, while microtubules did not appear to be involved in the process. The previously-undetected tubular PSV structures were characterized by complex movements, fusion events and a dynamic morphology. Their function during germination remains unknown, but might be related to the transport of solutes and metabolites.

## 1. Introduction

Seeds produce several different classes of storage proteins (prolamins, albumins and globulins) and diverse storage organelles protect these proteins from degradation [1,2]. In most plant species, protein storage vacuoles (PSVs) are the main storage organelles found in seeds [3,4,5,6]. They contain mostly albumins and globulins which typically pass through the Golgi body and accumulate in the PSV directly [1,2,7]. In cereal species, prolamins are produced as an additional class of storage proteins. Prolamins are often the most prominent storage proteins in cereal endosperm and are typically deposited into specialized, membrane-bounded storage organelles known as protein bodies (PBs). Depending on the species, PBs may remain in the cytoplasm as distinct, ER-derived organelles or eventually they may be incorporated into the PSVs [2,7]. Whereas PSVs are most abundant in seeds, the storage of metabolites, the digestion of cytoplasmic constituents and turgor maintenance in vegetative tissues are accomplished by central lytic vacuoles (LV) [8,9]. Different sets of cargo proteins and tonoplast intrinsic proteins (TIPs) have been routinely used to distinguish between the functionally different types of vacuoles [8,10], although it should be noted that some overlap of TIP markers has been observed upon recombinant expression [4]. Vacuolar development and remodeling during seed germination may involve the conversion of PSVs into LVs through cell type-specific sets of transformation events including PSV fusion, storage protein degradation, and the gradual replacement of TIPs [11,12].

The mature cereal grain is desiccated and is packed with reserves of starch, protein, lipids and minerals, providing the germinating seeds with energy and nutrients. The outer region of the endosperm comprises between one and three layers of epidermal aleurone cells, within which the PSVs are the most prominent organelles [13]. Aleurone PSVs primarily contain globulins [14] but prolamin-type storage proteins can also accumulate in the aleurone PSVs of certain species, including maize [15]. Aleurone PSVs also store small amounts of non-starch carbohydrates [16] and mineral phytate complexes that provide a source of phosphorus, potassium and magnesium [17]. Unlike starchy endosperm, the aleurone is viable in mature seeds. After imbibition, the highly differentiated and specialized aleurone tissue uses its stored reserves to synthesize and secrete digestive enzymes, which in turn mobilize the insoluble reserves in the starchy endosperm [14,18].

Germination is initiated by the imbibition of water. This causes the embryo to release gibberellic acid (GA_3_), which diffuses to the aleurone layer and induces the synthesis and secretion of hydrolases that degrade the starchy endosperm [18]. Expression profiling studies show that key transcripts required for the synthesis of GA_3_ are already strongly expressed in the endosperm and aleurone during grain maturation, and remain abundant during early germination (24 h after imbibition) [19]. In barley, it takes 4 days for the endosperm storage reserves to be depleted, and then the aleurone cells die. The storage products are then transported to the embryo via the scutellum.

Germination is also characterized by changes to the fine structure of aleurone cells [20]. De-embryonated half-grains, isolated aleurone layers and aleurone protoplasts have been used to study how cereal aleurone cells respond to hormones and to understand the molecular and cellular basis of signaling and regulation in the aleurone [18,21]. Barley aleurone protoplasts treated with GA_3_ secrete hydrolases that are synthesized *de novo* from amino acids released by the breakdown of storage proteins in PSVs [12,22]. The aleurone protoplasts then undergo dramatic morphological changes, in which the multiple small PSVs found in freshly isolated cells coalesce to form one large central vacuole after 4 days of GA_3_ treatment [20,23,24,25]. Secondary lytic vacuoles are found in barley aleurone protoplasts at the same time, and are possibly aleurain-containing vacuoles [26,27]. The vacuolation process correlates strongly with the duration of GA_3_ treatment and is complete after 5 days of incubation [21,28]. Vacuolation is linked to the dramatic conversion of nutrient-storing compartments into lytic organelles. Noninvasive measurements of the vacuolar pH in barley aleurone protoplasts has shown that the vacuole lumen pH in barley aleurone cells declines from 6.6 to 5.8 or below in a few hours in the presence of GA_3_ [25,27].

TIPs fused to fluorescent proteins are widely used as tonoplast markers for live cell imaging [4,29,30]. TIP3 (α-TIP) was first characterized as TP25, a seed-specific aquaporin that is strongly expressed in seeds but decreases rapidly during germination [31]. TIP3 is synthesized on the rough ER and appears to reach the tonoplast without passing through the Golgi body [31,32,33]. Expression assays and microscopy in Arabidopsis, barley aleurone protoplasts, and pea cotyledons confirmed that TIP3 is predominantly associated with PSVs in the seeds [4,10,27,29,32,34,35] and TIP3 has recently been used as a PSV (PBII) marker in rice subaleurone cells [30]. The ultrastructural changes of PSVs during barley endosperm maturation have been followed in situ by live cell imaging using a TIP3-GFP fluorescent tag [36]. Labelled PSVs in the subaleurone and central starchy endosperm cells underwent remarkable morphological changes, but the spherical PSVs in the aleurone layer did not change significantly [36]. The PSVs in the aleurone layer would be expected to undergo more profound changes during germination, but time-lapse imaging studies of living cells during germination have not been reported. We therefore used our established transgenic TIP3-GFP line [36] for the three-dimensional reconstruction and time-lapse analysis of PSV remodeling in barley aleurone cells during germination. Sections of intact germinating seeds were observed by confocal laser scanning microscopy (CLSM), showing that PSVs in barley aleurone cells undergo continuous remodeling, including a transition from spherical to ellipsoid to elongated morphology and complex processes of fusion, and possibly also scission and collapse. After 4 days of germination, highly mobile, tubular structures emerged from the PSVs, and their formation and mobility was promoted by actin microfilaments rather than microtubules. Potential functions of these TIP3-labeled tubular PSV extensions are discussed.

## 2. Results

### 2.1. Aleurone PSVs Become Larger and More Ellipsoid during Germination

**Figure 1:**
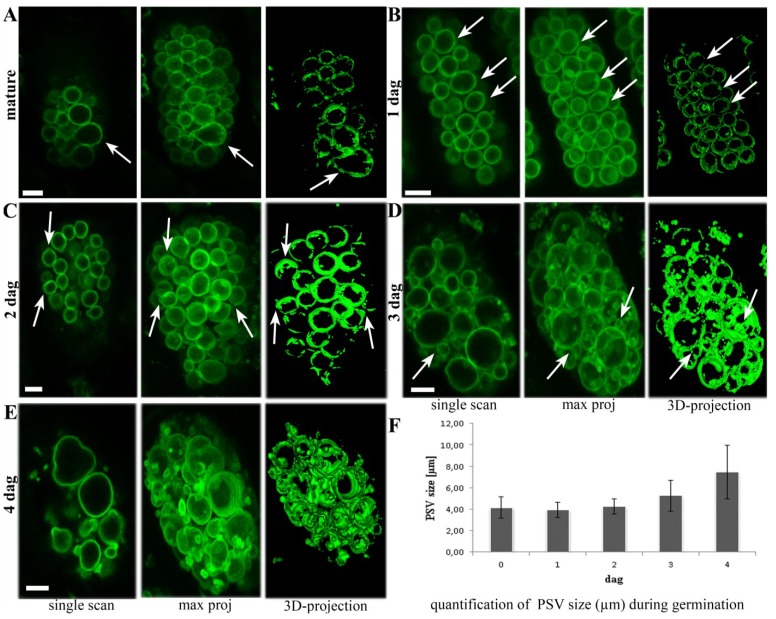
The dynamic morphology of tonoplast intrinsic proteins (TIP)3-GFP-labelled protein storage vacuoles (PSVs) during 4 days of germination. (**A**) TIP3-GFP labels spherical PSVs in the aleurone layer of mature kernels, plus occasional ellipsoid PSVs (arrows); (**B**) Homogeneous and tightly packed PSVs after one day of germination; (**C**) Arrows indicate PSVs starting to form buds at 2 days after germination; (**D**,**E**) PSVs become larger and more heterogeneous, with spherical, ellipsoid, small and large structures. Arrows indicate that small PSVs are still connected to large ones. Scale bar = 5 µm. (**F**) The average PSV size in µm during 4 days of germination. Standard error bars are indicated (n = 100).

The morphological changes that characterize PSVs in barley aleurone cells were studied by *in vivo* confocal microscopy, using homozygous T_4_ TIP3-GFP seeds up to 4 days after germination. At every time point, individual scans and 3D projections of z-series stacks were prepared for optimal visualization. In mature seeds, the PSVs in aleurone cells were strongly labeled with TIP3-GFP and appeared predominantly as spherical, discrete compartments (Figure 1A) although ellipsoid PSVs were occasionally observed (Figure 1A, arrow). This confirmed our previous study showing a similar labeling of aleurone PSVs during the late stages of endosperm development [36].

After one day of germination, the ellipsoid PSVs were more abundant, but the overall appearance of the PSVs remained homogenous and tightly packed (Figure 1B, arrows). Two days after germination, PSVs with buds were observed for the first time, indicating the beginning of the remodeling process (Figure 1C, arrows). Three days after germination, the morphological changes were more obvious, with the PSVs appearing more heterogeneous in shape and size (Figure 1D). Small PSVs appearing at this stage (Figure 1D, arrows) were shown to be connected to the larger ones in 3D projections, indicating that they probably result from reshaping and invagination processes (Figure 1D). Four days after germination, the TIP3-GFP-labeled PSVs underwent a more dramatic change in morphology, with few still spherical compartments but many more of the larger and more ellipsoid structures (Figure 1E). The average diameter of TIP3-labelled PSVs increased from 5 µm at 3 DAG (days after germination) to almost 8 µm a day later (Figure 1F). In addition, small TIP3-GFP-labeled PSVs appeared, only some of which were still connected to larger ones. In summary, live cell imaging provided a detailed overview of dynamic PSV morphology in the aleurone layer during 4 days of germination, showing that PSVs almost doubled in average size and changed from homogenous spherical to more diverse ellipsoid structures.

### 2.2. Real-Time Observations Indicate that Aleurone PSVs may Undergo Fusion, Scission and Collapse

**Figure 2:**
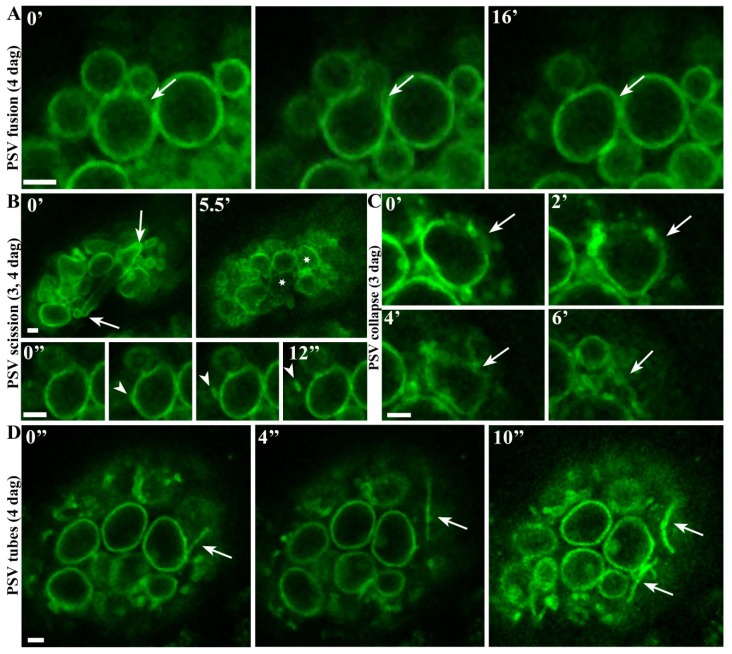
Live cell imaging 4 days after germination, indicating PSV fusion, scission and collapse, and the extension of tubular structures. (**A**) PSV fusion over a duration of 16 min (images captured every 4 s); (**B**) PSV apparently splitting into two PSVs (images captured every 8 s) and a TIP3-GFP-labeled vesicle budding off from another PSV (images captured every 4 s). (**C**) Potential PSV collapse over a duration of 6 min (images captured every 8 s). (**D**) Dynamic behavior of tubular extension structures (images captured every 4 s). Scale bar = 2 µm.

Live cell imaging also allowed us to follow the dynamic behavior of aleurone PSVs *in situ*. The most striking morphological changes took place 3–4 days after germination so time series were collected on these days by confocal microscopy over periods of 5–20 min. Figure 2A shows two PSVs of different sizes on day 4, fusing to form a larger PSV in 16 min (see also Movie 1). Potential scission events were also observed, including a large, narrow PSV (Figure 2B, arrows) apparently splitting into two smaller, spherical PSVs (Figure 2B, asterisks) in 5–6 min (Movie 2). A TIP3-GFP-labeled vesicle was also observed budding off from another PSV (Figure 2B, arrowheads) in 12 s. Occasionally, our observations indicated that PSVs were also collapsing (Figure 2C and Movie 3), although we cannot exclude the possibility that these observations might be attributed to other events giving the impression of collapse. Remarkably, we also observed highly mobile TIP3-GFP-labeled tubular structures at later stages of germination (Figure 2D and Movie 4). These structures were shown to appear, reshape, expand and retract again (Figure 2D arrows), predominantly 3–4 days after germination and usually at the periphery of the cells. Germination is therefore characterized by several dynamic events that affect aleurone PSVs, including fusion, remodeling and the generation of TIP3-GFP-labeled extensions that have not been reported thus far.

### 2.3. Highly Mobile Tubular Extension Structures Originate from PSVs

**Figure 3:**
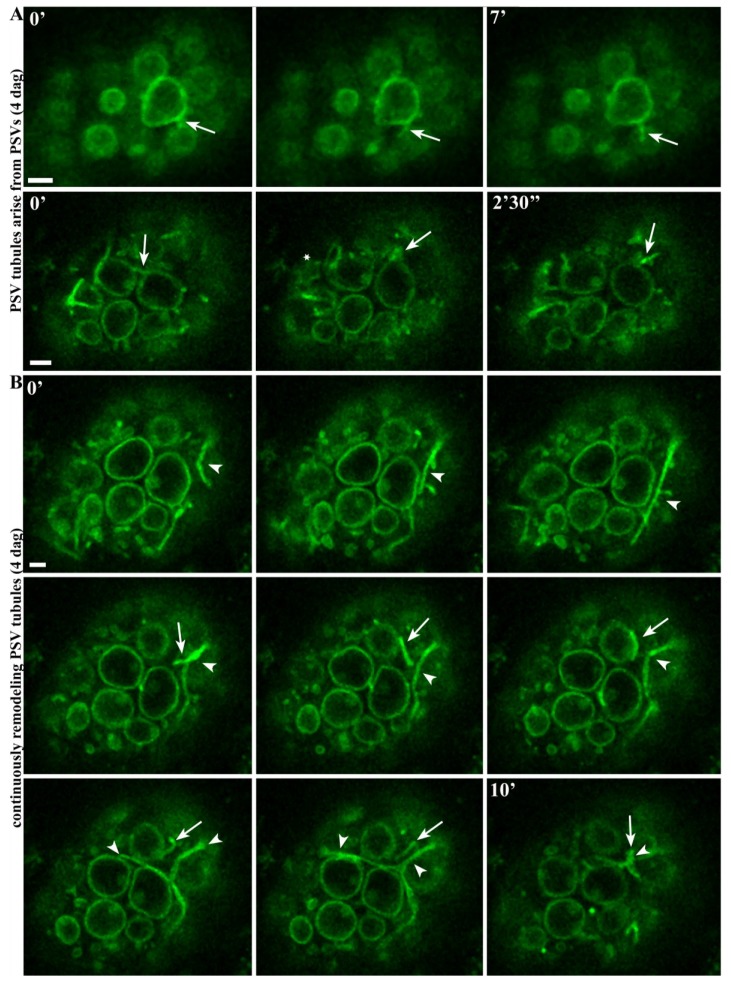
Confocal time series showing tubular structures arising from PSVs and undergoing continuous remodeling. (**A**) Time series of 7 and 2.5 min showing tubular structures arising from PSVs (images captured every 4 s). See the corresponding Movies 5 and 6 for more details; (**B**) Arrowheads and arrows show two different PSV extensions at the beginning of a 10-min time series (images captured every 4 s). See the corresponding Movie 7 for more details. Note the expansion and subsequent retraction of the structure, allowing the PSV extension to contact another PSV before release (arrow). Scale bar = 2 µm.

The dynamic events leading to the formation of TIP3-GFP-labeled tubular extensions were investigated by following the dynamic behavior of PSVs 3–4 days after germination by time-lapse confocal microscopy. The labeled structured first appeared 3 days after germination but became more prominent a day later. Two different time series were constructed, confirming that the TIP3-GFP-labeled tubules originate from PSVs (Figure 3A, arrows; Movies 5 and 6). Interestingly, we not only observed tubular extensions arising directly from the PSV surface, but another was formed by remodeling a connecting tube between two PSVs (Figure 3A and Movie 6).

The movement of the PSV tubules was tracked by time lapse imaging in a single cell (Figure 3B and Movie 7). These images show that the tubular PSV structure elongates to double its original length, retracts again and then realigns (Figure 3B, arrowhead). An adjacent loop in the same cell contracts and aligns with another PSV membrane, where it is released. At the same time, the original PSV tube expands and contracts again (Figure 3B, arrowheads; Movie 7). Several tubules also expand, contact each other, separate, and new ones are generated (Movie 7). These data show that the PSV tubules are constantly remodeling, coming into contact with each other and with other PSVs. The speed of the movement was 0.1–0.2 µm/s.

### 2.4. The Formation of PSV Tubules Depends on Microfilaments but not Microtubules

**Figure 4:**
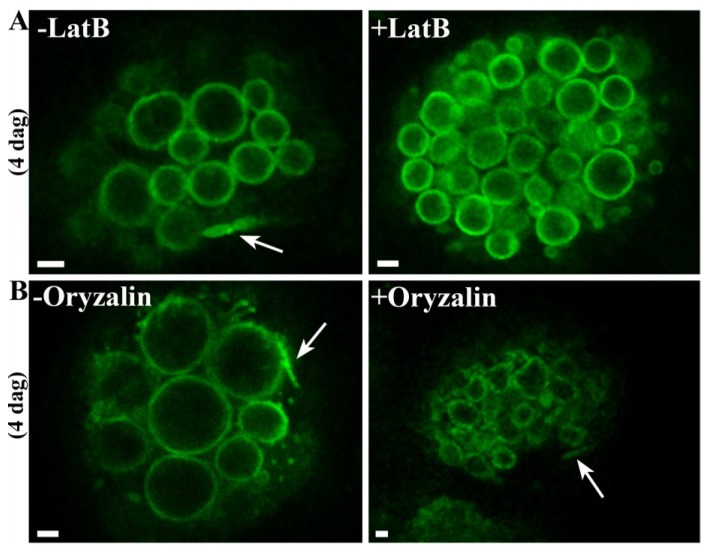
The remodeling of tubular PSV extensions depends on microfilaments but not microtubules. (**A**) Representative pictures from movies showing PSV tubules in untreated cells (arrow) but no PSV tubules in cells treated with latrunculin B; (**B**) Representative pictures showing PSV tubules in untreated cells (arrow) and in cells treated with oryzalin. Scale bar = 2 µm.

The cytoskeleton is generally important for vacuolar transport and remodeling, so we specifically inhibited the activity of microfilaments or microtubules during germination to determine their role in the dynamic behavior of PSVs. Seeds were soaked twice, first at the mature stage and then at 2 days after germination, with 10 µM latrunculin B (to inhibit microfilaments) or 10 µM oryzalin (to inhibit microtubules). Both chemicals inhibited germination substantially, whereas the equivalent working concentration of DMSO (the solvent used for the stock solutions) had no effect [37]. Observations by confocal microscopy 4 days after germination showed that seeds incubated with latrunculin B did not produce PSV tubules (Figure 4A) whereas seeds treated with oryzalin produced dynamic PSV extensions like those observed in untreated cells (Figure 4B). These data indicate that the formation of PSV tubules is actin-dependent. Notably, neither latrunculin B nor oryzalin had a significant effect on the underlying PSV morphology during germination.

## 3. Discussion

The endomembrane system of cereal seeds undergoes a series of developmental changes during maturation and germination, first to accomplish the deposition of storage compounds and then their mobilization, both within a relatively short period of time. Germination takes only a few days, during which the cells of the aleurone layer deplete their reserves stored in PSVs. The ultrastructural changes of aleurone organelles during germination have been investigated previously mainly by static imaging techniques, often using fixed cells from de-embryonated half-grains, isolated aleurone layers or aleurone protoplasts treated with GA_3_ [12,21]_._ There have been only few reports describing the analysis of endomembrane organelles in living cereal seeds, partly reflecting the limited availability of transgenic lines containing fluorescent organelle markers.

**Figure 5:**
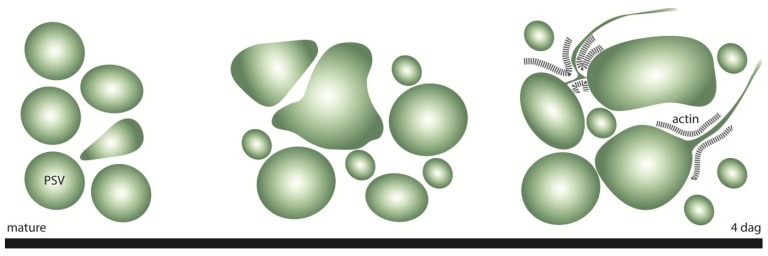
Schematic summary of the morphological changes of the PSVs and the formation of PSV tubules in barley aleurone cells during germination. dag = days after germination.

We therefore used a transgenic barley line expressing a fluorescent TIP3-GFP fusion protein to visualize the PSV and carried out detailed *in vivo* studies including time-lapse imaging, z-series and 3D projections. These studies revealed the remarkably dynamic morphology of PSVs during germination (Figure 5). The homogeneous spherical PSVs present in the mature seed changed progressively to larger, ellipsoid structures over 4 days of germination.

Unlike previous studies using isolated aleurone layers and protoplasts treated with GA_3_ [20,23,24,25], we did not observe a single large central vacuole at the end of germination. It is therefore likely that interaction and communication among the aleurone, subaleurone and starchy endosperm cells contribute to the regulation of germination and particularly the behavior of storage organelles. The aleurone and scutellum are controlled by plant hormones such as GA_3_ and ABA [19,25,27,38] but may also be influenced by feedback from the mobilized reserved released from starchy endosperm cells [18]. ABA antagonizes the events induced by GA_3_ in the aleurone at many levels, suggesting there is a sensitive regulatory system based on the balance between these two hormones [18] as well as processes regulated by auxins and ethylene [19]. Although the same cellular events occur in the aleurone of whole imbibed grain and GA_3_-treated aleurone layers and protoplasts, the events seem more rapid in isolated aleurone layers and protoplasts, perhaps because the direct application of single hormones overwhelms the endogenous interaction network [21] and eliminates interactions based on the arrangement of tissues and the macromolecules stored during development [18]. This is consistent with our observation that PSVs in aleurone cells near to the starchy endosperm behave in a different manner to PSVs near the testa [37]. The PSVs in the aleurone layer adjacent to the starchy endosperm are larger, indicating that PSV fusion is favored in these cells.

The fusion of the PSVs into larger vacuolar structures is a common feature that has been observed in both monocotyledonous and dicotyledonous plants with seed storage tissues that are still alive at the point of reserve mobilization [14]. This includes embryo cotyledon and root tissues, castor bean endosperm cells and cereal aleurone layers [11,12,39]. By focusing on living aleurone cells containing TIP3-GFP-labeled PSVs, we were able to monitor in real time the PSV remodeling processes throughout germination. Our time-lapse series revealed the dynamic processes underlying the observed morphological changes, including vacuole fusion and potential fission events and the deformation of initially spherical PSVs to produce ellipsoid or irregular structures that budded off smaller PSVs. Three-dimensional reconstructions revealed that connections were maintained between some small PSVs and the larger ones, whereas others were independent and spherical. Vacuoles also appeared to collapse occasionally, perhaps reflecting tonoplast destabilization at the onset of programed cell death [21].

Most conspicuously, on the third and fourth days of germination we observed TIP3-GFP-labeled, constantly remodeling tubular structures that arose directly from the PSVs. Tubular vacuoles in plant cells were first observed in 1929 in rose leaves, and were further characterized in onion epidermal and guard cells, and soybean root cells [40,41,42]. Such structures are often associated with specialized cell types including root hairs [43] and pollen tubes [44]. Tubular vacuoles extending from the large central vacuole were described in great detail in red onion epidermal cells [45]. More recently, unique tubular structures were observed using a photoconvertible mEosFP::2xFYVE probe, which labeled putative endosomes, prevacuoles and vacuoles [46]. The structures were described as aberrant oscillating or rotating vesicles that extended tubular projections 5–15 µm in length and ~0.6 µm in diameter. Time-lapse sequences showed that structures were generated constantly, extending from different vesicles, forming loops and snaring other vesicles into loose aggregates. The ends of the observed tubules were shown to be sealed rather than forming a continuous compartment [46]. The tubular structures we observed were similar in size and mobility, and likewise did not form a continuous network. Membrane protrusions have also been observed to various degrees for other organelles. For example, in plastids, dynamic tubular extrusions of the inner and outer envelopes, termed stromules, have been described [47,48]. Since stromules increase the surface area of plastids it has been speculated that they might facilitate the interaction and the exchange of metabolites with other plastids, mitochondria, peroxisomes and the endoplasmic reticulum [49,50,51,52].

The functional role of vacuolar tubules remains unclear. They may play a role in the transport of sugars, other nutrients and metabolites in fungi and yeast cells [53,54]. Therefore, the dynamic PSV tubules we observed in barley aleurone cells during germination may also transfer reserves or other materials between PSVs. It is also possible that the role of the PSV extensions may be linked to an increased rate of transport between the cytoplasm and vacuolar lumen due to the expansion of the tonoplast surface.

Alternatively, the PSV tubules may lack a specific function and may instead represent a stage in vacuolar development [55]. Tubular intermediate structures are involved in the transformation of PSVs into lytic vacuoles in the root cells of tobacco seedlings during germination and early root development [11]. Based on TEM studies combined with high-pressure freezing and freeze-substitution techniques, it has been suggested that mobilization of storage molecules induces the gradual osmotic collapse of the vacuolar membranes upon themselves, thereby squeezing the residual vacuolar contents into the remaining bulging vacuolar regions [11]. However, the PSV tubules we observed appear later in the process, following the bulging vacuolar regions that were pictured during the mobilization of storage reserves. However, the duration and individual steps of the vacuolar transition process are highly variable depending on the cell type [11]. Therefore, it will be necessary to determine the content and pH of the PSV tubules in order to clarify their role during barley germination. It is notable that we did not observe the tubular structures when tracking PSVs in the same barley line during endosperm maturation, suggesting they are specific for germination and do not represent artifacts induced by laser scanning microscopy or the expression of TIP3 as a vacuolar marker [36].

Time-lapse imaging after the treatment of germinating seeds with the microfilament inhibitor latrunculin B or the microtubule inhibitor oryzalin indicated that the formation of PSV extensions depends on microfilaments but not on microtubules. Similar results have been reported in lily pollen tubes, where vacuolar morphology and the movement of long tubular vacuolar structures are significantly affected by latrunculin B but not oryzalin [56]. The dependence of higher plant vacuolar tubules on actin and myosin has also been reported in other systems [43,57] and is consistent with the actin-based motility of vesicle trafficking to the central vacuole [58,59]. In contrast to previous studies showing the inhibition of vacuolar tubule movement, latrunculin B prevented the formation of PSV tubules in barley aleurone in the first place, agreeing with reports indicating that actin-interacting proteins may be involved in the formation of intravacuolar structures and in vesicle formation and/or release [58,60]. In the absence of actin filaments, proteins needed for vesicle formation and/or release (e.g., dynamins, Rab GTPase) may not be efficiently recruited, thus inhibiting reshaping events [45,61]. Actin filaments might also affect the generation and dynamic behavior of the PSV tubules indirectly by inhibiting cytoplasmic streaming, if the induction and dynamic behavior of the PSV extensions were influenced by the bulk flow of the cytoplasm [45]. Further studies are needed to clarify the role of actin filaments in the formation and expansion of PSV tubules and such studies will also help to determine the function of these novel structures.

## 4. Experimental

### 4.1. Plant Material

Barley wild-type (Golden Promise) and T4 homozygous TIP-GFP transgenic seeds [36] were plated on filter paper soaked in tap water and incubated for 4 days in Petri dishes at room temperature. Mature seeds were soaked for a maximum of 2 h in tap water.

### 4.2. Confocal Microcopy

Samples were mounted within a small border of Vaseline to avoid floating, and silicon glue was used to stabilize the cover slips. GFP images were captured using a Leica SP5 CLSM with excitation and emission wavelengths of 488 and 500–530 nm. Transgenic homozygous T_4_ TIP3-GFP barley seeds at different germination stages (mature to 4 days after initiating germination) were sectioned, washed, mounted in tap water and analyzed immediately. Images were processed using ImageJ and arranged using Photoshop CS5. Movies are displayed at 17 frames per second.

### 4.3. Calculation of PSV Size

The average diameter of PSVs (n = 100) was calculated at different stages ranging from mature seeds to 4 days after germination, by measuring the length of the maximum distance between opposite membranes of the spherical and irregular PSVs.

### 4.4. Inhibitor Studies

Homozygous T_4_ TIP3-GFP seeds were germinated in 24-well plates for 4 days. Dry seeds were placed in 400 µL of solution containing 10 µM latrunculin B or 10 µM oryzalin in tap water. The solutions were replaced with fresh ones after two days of germination. Each solution was prepared from a 1 mM stock solution in DMSO, and the control was therefore prepared by diluting pure DMSO to the same working concentration in tap water. Sections were mounted in tap water and analyzed immediately for a maximum of 1 h by CLSM as above. For each experiment, at least four seeds were sectioned at the appropriate germination stage to determine the effect of the inhibitor.

## 5. Conclusions

Live cell imaging has allowed us to follow in real time the morphological changes of PSVs in the aleurone cells of germinating transgenic barley seeds expressing a fluorescent PSV marker. This process has been studied before, but only by reconstructing static images, mostly based on fixed tissues and cells. Our live cell imaging method confirmed that PSVs undergo spatiotemporal remodeling during barley germination. Remarkably, during the late stages of germination we observed thin tubules extending from PSVs in an actin dependent mode. These previously unreported structures underwent complex movements, comprising fusion events and dynamic changes in morphology. The function of the PSV tubules remains unclear but may be related to the transport of reserves between PSVs or from the PSV to the cytoplasm. Alternatively, they may represent a stage in vacuolar development heralding the onset of programed cell death. Additional investigations are required to determine the content and pH of the PSV tubules and the precise mechanism by which actin filaments control their formation, extension, retraction and interactions, thus providing further insight into their function during germination.

## Supplementary Files

Supplementary File 1
